# Impact of the COVID-19 pandemic on the care and outcomes of people with NAFLD-related cirrhosis

**DOI:** 10.1016/j.jhepr.2022.100574

**Published:** 2022-08-27

**Authors:** Jesús Rivera-Esteban, Ramiro Manzano-Nuñez, Teresa Broquetas, Isabel Serra-Matamala, Octavi Bassegoda, Agnès Soriano-Varela, Gemma Espín, Joaquín Castillo, Juan Bañares, José A. Carrión, Pere Ginès, Isabel Graupera, Juan M. Pericàs

**Affiliations:** 1 Liver Unit, Vall d’Hebron University Hospital, Barcelona, Spain; 2 Vall d’Hebron Institut de Recerca (VHIR), Vall d’Hebron Barcelona Campus Hospitalari, Barcelona, Spain; 3 Universitat Autònoma de Barcelona, Barcelona, Spain; 4 Liver Section, Gastroenterology Department, Hospital del Mar, Barcelona, Spain; 5 IMIM (Hospital del Mar Medical Research Institute), Barcelona, Spain; 6 Department of Medicine and Life Sciences, Universitat Pompeu Fabra, Barcelona, Spain; 7 Centro de Investigación Biomédica en Red de enfermedades digestivas y hepáticas (CIBERehd), Madrid, Spain; 8 Dr Josep Trueta University Hospital, Girona, Spain; 9 Liver Unit, Hospital Clínic de Barcelona, IDIBAPS, University of Barcelona, Barcelona, Spain

**Keywords:** Nonalcoholic fatty liver disease, cirrhosis, COVID-19, health systems, liver outcomes, cACLD, compensated advanced chronic liver disease, HCC, hepatocellular carcinoma, NAFLD, non-alcoholic fatty liver disease, OR, odds ratio, T2D, type 2 diabetes, VCTE, vibration-controlled transient elastography

## Abstract

**Background & Aims:**

The COVID-19 pandemic has had a major negative impact on health systems and many chronic diseases globally. We aimed to evaluate the impact of the first year of the pandemic on the outcomes of people with NAFLD cirrhosis.

**Methods:**

We conducted a before-after study in four University hospitals in Catalonia, Spain. Study subperiods were divided into Pre-pandemic (March/2019–February/2020) *vs.* Pandemic (March/2020–February/2021). The primary outcome was the rate of first liver-related event (LRE). Overall clinical outcomes (LREs plus cardiovascular plus all-cause mortality) were also assessed.

**Results:**

A total of 354 patients were included, all of whom were compensated at the beginning of the study period; 83 individuals (23.5%) had a history of prior hepatic decompensation. Mean age was 67.3 years and 48.3% were female. Median BMI was 31.2 kg/m^2^ and type 2 diabetes was present in 72.8% of patients. The rates of first LRE in the Pre-pandemic and Pandemic periods were 7.4% and 11.3% (*p* = 0.12), respectively. Whilst the rate of overall events was significantly higher in the Pandemic period (9.9% *vs.* 17.8%; *p =* 0.009), this was strongly associated with COVID-19-related deaths. The rate of worsened metabolic status was significantly higher in the Pandemic period (38.4% *vs.* 46.1%; *p =* 0.041), yet this was not associated with the risk of first LRE during the Pandemic period, whereas type 2 diabetes (odds ratio [OR] 3.77; 95% CI 1.15–12.32; *p =* 0.028), albumin <4 g/L (OR 4.43; 95% CI 1.76–11.17; *p =* 0.002) and Fibrosis-4 score >2.67 (OR 15.74; 95% CI 2.01–123.22; *p =* 0.009) were identified as risk factors in the multivariable analysis.

**Conclusion:**

Overall, people with NAFLD cirrhosis did not present poorer liver-related outcomes during the first year of the pandemic. Health system preparedness seems key to ensure that people with NAFLD cirrhosis receive appropriate care during health crises.

**Lay summary:**

Mobility restrictions and social stress induced by the COVID-19 pandemic have led to increased alcohol drinking and worsened metabolic control (*e.g*., weight gain, poor control of diabetes) in a large proportion of the population in many countries. We aimed to analyze whether people with cirrhosis due to non-alcoholic fatty liver disease, who are particularly vulnerable to such lifestyle modifications, were significantly impacted during the first year of the pandemic. We compared the clinical situation of 354 patients one year before the pandemic and one year after. We found that although metabolic control was indeed worse after the first year of the pandemic and patients presented worse clinical outcomes, the latter was mostly due to non-liver causes, namely COVID-19 itself. Moreover, the care provided to these patients did not worsen during the first year of the pandemic.

## Highlights

• Patients with NAFLD cirrhosis did not present a higher rate of liver-related events during the COVID-19 pandemic.
• Usual predictors, such as diabetes, albumin and FIB-4 were associated with higher risk of a first liver event.
• Health system preparedness seems key to ensure patients with NAFLD cirrhosis receive appropriate care during health crises.

## Graphical abstract

**Figure:**
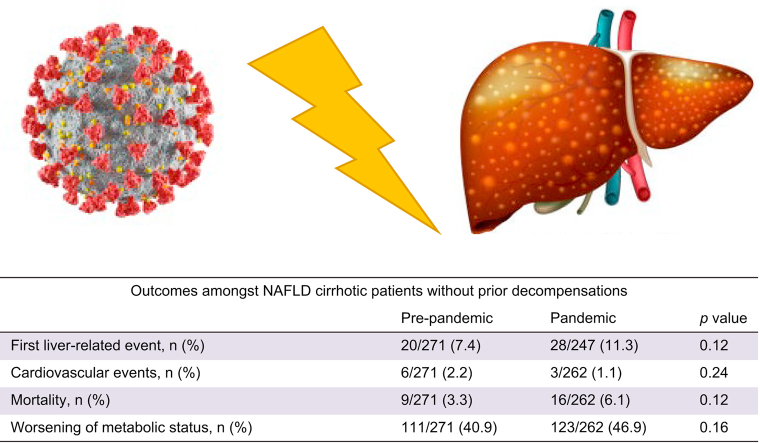

## Introduction

The COVID-19 pandemic has had a strong, overall negative impact on health systems globally in terms of patient suffering, healthcare overloads, and economic burden.^1^ A specific impact was in the tertiary setting, particularly during the first wave, when healthcare resources were reassigned to COVID-19 and routine care was deferred for ‘stable’ patients to mitigate the spread of severe acute respiratory syndrome coronavirus 2 (SARS-CoV-2). Hence, many other specialties (*i.e*., hepatology units) suffered from a diversion and reduction of their resources that affected the delivery and quality of care.[2], [3], [4], [5], [6]

People with chronic liver disease, including non-alcoholic fatty liver disease (NAFLD), are at higher risk of severe COVID-19, disease progression and clinical decompensation^.^[7], [8], [9], [10], [11], [12], [13], [14], [15], [16] Moreover, lockdown, economic hardship and the psychological impact of the pandemic all had a detrimental effect on people with liver disease, including poorer metabolic control in people with metabolic syndrome and fatty liver disease.^17^^,^^18^ This likely had deleterious consequences on the liver and cardiovascular (CV) outcomes of people with NAFLD, particularly those with advanced liver disease. Mortality has been shown to increase in people with alcohol-associated liver disease,^19^ and there have also been several reports on the impact of the COVID-19 pandemic on the diagnosis and management of hepatocellular carcinoma (HCC),[20], [21], [22] cirrhosis,[23], [24], [25], [26] and patients requiring liver transplantation.^27^

However, the impact of the pandemic on people with cirrhosis due to NAFLD is poorly known. Therefore, in this study, we aimed to evaluate how the effect of COVID-19 on health systems during the first wave of the pandemic impacted outcomes in people with NAFLD cirrhosis.

## Patients and methods

### Design and setting

We conducted a multicentric before-after study based on NAFLD cohorts with retrospective data from four university hospitals in Barcelona (three) and Girona (one), Catalonia, Spain. The study period encompasses the period from March 2019 to March 2021 and has been divided into two subperiods: the year before the Spanish government declared the state of emergency (March 2019 – February 2020; Pre-pandemic period), and the year after that (March 2020 – February 2021; Pandemic period).

In the four participating hospitals, staff of hepatology units were assigned to COVID-19 clinical tasks at least during the first wave (March-May 2020) of the pandemic, and in some cases also in latter outbreaks. However, in all hospitals biannual visits were kept for people with cirrhosis; liver and metabolic changes were recorded; and blood tests and abdominal ultrasound schedules were maintained. During COVID-19 peaks, in-person visits were replaced by video calls or telephone calls. In decompensated patients, either admitted to the hospital or not, the frequency of follow-up calls was increased. In brief, the recommendations included in the EASL-ESCMID position paper on the care of people with liver disease during the COVID-19 pandemic^2^ were followed.

### Participants

People with a diagnosis of cirrhosis due to NAFLD before March 2019 under follow-up at liver clinics of the participating hospitals were included.

### Definitions

*NAFLD cirrhosis*: one or more of the following criteria: liver biopsy with ≥5% steatosis and/or steatohepatitis by NASH clinical research network score^28^ and fibrosis stage 4 or cryptogenic cirrhosis in a patient with known obesity, type 2 diabetes (T2D) or metabolic syndrome and no other detectable liver etiology; presence of steatosis on imaging and signs of ultrasonographic or endoscopic portal hypertension in a patient with compensated advanced chronic liver disease (cACLD) and obesity, T2D or metabolic syndrome in the absence of other etiologies of cACLD (signs of ultrasonographic portal hypertension were the presence of splenomegaly [>13 cm], portal-systemic collaterals, inversion of flow within the portal system, dilatation of portal vein [diameter >13 mm] or reduced portal vein velocity <10 cm/s); presence of steatosis on imaging and liver stiffness ≥18 kPa by vibration-controlled transient elastography (VCTE) in a patient with obesity, T2D or metabolic syndrome in the absence of other etiologies of cACLD. Of note, no other imaging technique different from VCTE was used for liver fibrosis estimation.

*First liver event*: first episode of ascites of any grade (stage 1 to 3), any grade of hepatic encephalopathy (HE) according to the West-Haven classification (stage 1 to 4), portal hypertension-related bleeding, or hepatocellular carcinoma in people with compensated cirrhosis.

*Liver events*: portal hypertension-related bleeding, any grade of HE, or ascites, spontaneous bacterial peritonitis (in people with refractory ascites), hepatocellular carcinoma, and liver transplant.

*Cardiovascular events*: acute coronary syndrome, acute stroke, others (*e.g.*, acute peripheral arterial syndrome).

*Weight gain*: any measured weight gain compared to one year earlier (under the assumption that people with NAFLD are supposed to lose weight or maintain it); *Significant body weight gain*: >5%.

*Poor control of diabetes*: new diagnosis of T2D and/or fasting glucose >140 and/or Hb1Ac >8%, and/or introduction of new drug to treat T2D.

*Poor control of systemic hypertension*: new diagnosis of high blood pressure and/or routine measurements of systolic arterial pressure >140 mmHg or diastolic arterial pressure >90 mmHg and/or episodes of hypertensive crisis-emergencies, and/or new drug added.

*Poor control of dyslipidemia*: new diagnosis of dyslipidemia (either due to hypercholesterolemia, hypertriglyceridemia or both) and/or total cholesterol >240 mg/dl and/or total triglycerides >200 mg/dl, and/or new drug added.

*Worsening of metabolic status*: Presence of at least one of the previous variables (significant weight gain and/or poor control of diabetes mellitus/arterial hypertension/dyslipidemia).

*Delayed diagnosis of HCC*: >2 months after an imaging test was performed suggesting HCC.

*Delayed treatment of esophageal varices*: >2 months after a gastroscopy showing new or advanced changes requiring new or additional treatment (either endoscopic or pharmacological).

### Outcomes

The primary outcome was the development of clinical events during the study period, particularly a first liver-related event (LRE) amongst persons without prior decompensations. A first LRE was defined as the development of a clinical decompensation (ascites, hepatic encephalopathy, or upper gastrointestinal bleeding secondary to portal hypertension) or HCC.

As secondary outcomes, we investigated: the occurrence of overall clinical events (hepatic, also including spontaneous bacterial peritonitis, and CV), liver-related and all-cause mortality in the entire study cohort (including both persons with and without a prior decompensation at the beginning of the study period); worsening of metabolic status; and delay of management of cirrhosis complications (HCC diagnosis and endoscopic treatment of esophageal varices).

### Ethics

Vall d’Hebron University Hospital Campus IRB approved the study protocol (code PR(AG)461/2021). All patients provided informed consent.

### Statistical analyses

Continuous variables are presented as means (SD) or medians (IQR) as appropriate. Categorical variables are presented as frequencies and percentages.

Primary and secondary outcomes were compared for two periods: Pre-pandemic *vs.* Pandemic. Continuous variables were compared using paired *t* tests or the Wilcoxon matched-pairs signed-rank test, according to the normality of their distribution. On the other hand, categorical variables were compared by performing tests on the equality of proportions.

Incidence rate ratios were estimated for the Pandemic period and compared with the Pre-pandemic period using indicator variables. A logistic regression analysis was performed to identify risk factors associated with the development of a first LRE during the Pandemic period. We graphed Kaplan-Meier survival curves for the first LRE and a log-rank test was performed. All analyses were performed in Stata 13.1 Statistical Software (StataCorp, College Station, TX, USA).

## Results

### Sample

The study cohort comprised 354 persons with compensated NAFLD cirrhosis, 271/354 (76.5%) of whom had no history of decompensation, while 83 individuals (23.5%) had presented with a prior episode(s) of hepatic decompensation. Individuals with a Child-Pugh A score represented 86.9% of the sample, while those with Child-Pugh B and C represented 12.1% and 1%, respectively. The diagnosis of NAFLD cirrhosis was established by liver biopsy in 106 patients (35.6%), whereas 103 (29.1%) and 125 (35.3%) individuals were classified as having cirrhosis based on liver stiffness ≥18 kPa by VCTE or signs of portal hypertension, respectively. Of note, median follow-up time from the diagnosis of NAFLD cirrhosis was 2.54 years (IQR 1.23–5.13).

### Baseline characteristics

Table 1 shows the main characteristics from the entire cohort. Mean age was 67.3 years (SD 9.6) and 48.3% were female. Seventy-six patients (21.5%) presented non-harmful alcohol consumption and 17.7% were active smokers. Median BMI was 31.2 kg/m^2^ (IQR 27.6–35.1) and 57.8% were obese (BMI ≥30 kg/m^2^). T2D was present in 72.9% of patients, while 70.9% and 51.1% had arterial hypertension and dyslipidemia, respectively. At baseline, 87% of patients were classified as Child-Pugh A.

**Table 1:** **Baseline characteristics of 354 people with compensated NAFLD cirrhosis included in the study**.

	Overall n = 354	Without prior decompensations n = 271	With prior decompensations n = 83	*p* value
Age, mean years (SD)	67.3 (9.6)	66.9 (9.2)	68.8 (10.5)	0.11
Females, n (%)	171 (48.3)	132 (48.7)	39 (47.0)	0.78
Tobacco use, n (%)	38 (10.8)	27 (10.1)	11 (13.3)	0.41
Alcohol use, n (%)∗	76 (21.5)	60 (22.1)	16 (19.3)	0.57
BMI, median kg/m^2^ (IQR)	31.2 (27.6-35.1)	31.8 (27.8-35.3)	30.2 (26.9-32.9)	0.023
BMI ≥25 kg/m^2^, n (%)	302 (92.9)	240 (94.1)	62 (88.6)	0.10
BMI ≥30 kg/m^2^, n (%)	188 (57.8)	150 (58.8)	38 (54.3)	0.49
Arterial hypertension, n (%)	251 (70.9)	193 (71.2)	58 (69.9)	0.81
T2D, n (%)	258 (72.9)	196 (72.3)	62 (74.7)	0.67
Dyslipidaemia, n (%)	181 (51.1)	142 (52.4)	39 (47.0)	0.38
Previous stroke, n (%)	16 (4.5)	14 (5.2)	2 (2.4)	0.29
Previous ischemic heart disease, n (%)	35 (9.9)	26 (9.6)	9 (10.8)	0.73
Child-Pugh score				
A/B/C, n (%)	287 (86.9)/40 (12.1)/3 (1.0)			
Liver stiffness, mean kPa (SD)∗∗	23.6 (14.8)	22.9 (13.8)	33.9 (25.4)	0.10
CAP, mean dB/m (SD)∗∗∗	307.0 (58.0)	308.1 (57.6)	281.3 (77.6)	0.43

Hypertension: ≥140/90 mmHg or requiring treatment; type 2 diabetes: as a fasting plasma glucose ≥126 mg/dl or a non-fasting plasma glucose ≥180 mg/dl or requiring treatment.; dyslipidemia: serum triglycerides ≥150 mg/dl and/or total cholesterol >200 mg/dl, LDL >130 mg/dl, HDL<40 mg/dl in men and <50 mg/dl in women or requiring treatment.

Continuous variables were compared using *t* test or Wilcoxon rank-sum, depending on the normality of their distribution. Categorical variables were compared using the chi-square test. A *p* <0.05 was considered statistically significant.

CAP, controlled attenuation parameter; T2D, type 2 diabetes.

∗ Alcohol intake was defined as <20 g/day and <30 g/day for women and men, respectively.

∗∗ Data available in 83 individuals.

∗∗∗ Data available in 75 individuals.

Individuals with and without prior decompensations showed similar demographic and metabolic comorbidity rates, including overweight and obesity prevalence. Mean values of VCTE (liver stiffness and controlled attenuation parameter) were also comparable between groups.

### Comparison before and after the pandemic outbreak

As shown in Table 2, platelet count, bilirubin, and renal function worsened during the Pandemic period in the overall cohort. Accordingly, Fibrosis-4 (FIB-4) and model for end-stage liver disease score showed higher values after the outbreak of the pandemic. Median time between blood analyses was 17.1 months (IQR 12.4–20.1). No changes were observed regarding transaminase levels, mean glucose or lipid profile. Paired individual before-after VCTE data were available in only 10.1% of the overall study cohort. No differences were found in liver stiffness and controlled attenuation parameter values between study periods.

**Table 2:** **Changes in biochemical and non-invasive tests before and after the outbreak of the COVID-19 pandemic**.

	Overall, n = 354			Without prior decompensations, n = 271			With prior decompensations, n = 83		
	Pre-pandemic	Pandemic	*p* value	Pre-pandemic	Pandemic	*p* value	Pre-pandemic	Pandemic	*p* value
Platelets (10^9^/L)	143 (68)	138 (67)	0.02	152 (70)	148 (68)	0.13	111 (50)	103 (50)	0.16
INR	1.15 (0.25)	1.17 (0.38)	0.33	1.14 (0.27)	1.14 (0.35)	0.94	1.19 (0.17)	1.27 (0.44)	0.07
Glucose (mg/dl)	141.1 (60.9)	146.7 (65.9)	0.25	143.9 (66.8)	148.3 (71.7)	0.48	132.5 (37.3)	142.1 (44.8)	0.19
Creatinine (mg/dl)	0.91 (0.47)	0.96 (0.57)	0.002	0.87 (0.35)	0.91 (0.48)	0.042	1.06 (0.73)	1.16 (0.79)	0.004
Bilirubin (mg/dl)	0.94 (0.55)	1.06 (1.21)	0.022	0.86 (0.48)	0.91 (0.53)	0.019	1.22 (0.69)	1.63 (1.34)	0.09
AST (U/L)	38.7 (17.8)	39.3 (22.2)	0.61	38.0 (17.5)	37.8 (18.9)	0.82	41.3 (18.7)	44.7 (31.0)	0.35
ALT (U/L)	34.7 (22.8)	33.4 (22.7)	0.32	35.2 (21.3)	33.4 (21.0)	0.14	33.2 (27.8)	33.3 (28.4)	0.98
Total CT (mg/dl)	166.3 (39.1)	168.1 (41.6)	0.3	168.0 (38.9)	171.4 (39.8)	0.054	159.9 (39.4)	155.8 (45.9)	0.41
HDL (mg/dl)	49.5 (17.2)	51.0 (17.9)	0.24	49.3 (17.4)	51.4 (17.9)	0.09	51.6 (15.5)	47.6 (18.1)	0.34
LDL (mg/dl)	92.8 (34.8)	92.9 (34.0)	0.95	92.9 (33.9)	93.3 (33.3)	0.82	92.4 (42.5)	90.0 (39.8)	0.84
Triglycerides (mg/dl)	141.5 (94.8)	142.1 (85.9)	0.88	147.3 (100.4)	150.7 (92.0)	0.47	120.1 (66.8)	110.3 (46.3)	0.13
Albumin (g/dl)	4.10 (0.52)	4.07 (0.58)	0.14	4.20 (0.48)	4.17 (0.53)	0.25	3.76 (0.53)	3.71 (0.64)	0.36
FIB-4 score	4.16 (2.93)	4.57 (3.82)	0.003	3.72 (2.51)	4.00 (3.29)	0.047	5.74 (3.71)	6.64 (4.79)	0.026
MELD score	7.67 (3.00)	8.16 (4.01)	0.001	7.36 (2.70)	7.55 (3.11)	0.12	8.77 (3.69)	10.35 (5.77)	0.001
Liver stiffness (kPa)∗	22.3 (13.5)	22.8 (16.6)	0.84						
CAP (dB/m)∗∗	315.0 (61.8)	293.8 (85.2)	0.14						

Data are presented as mean (SD). Continuous variables were compared using paired *t* tests or the Wilcoxon matched-pairs signed-rank test, according to the normality of their distribution. On the other hand, categorical variables were compared by performing tests on the equality of proportions. A *p* <0.05 was considered statistically significant.

ALT, alanine aminotransferase; AST, aspartate aminotransferase; CAP, controlled attenuation parameter; CT: cholesterol; FIB, Fibrosis-4; INR, international normalized ratio; MELD, model for end-stage liver disease.

∗ Data available in 35 individuals.

∗∗ Data available in 30 individuals.

### Clinical outcomes in people with compensated NAFLD cirrhosis without prior decompensations

During the Pandemic period, 28 individuals (11.3%) presented a first hepatic event compared to 7.4% (20/271) before the pandemic outbreak (*p =* 0.12). The most frequent liver event was ascites in both periods (Table 3).

**Table 3:** **Clinical outcomes amongst people with NAFLD cirrhosis without prior decompensations**.

Outcomes	Pre-pandemic	Pandemic	*p* value
First liver-related event, n (%)	20/271 (7.4)	28/247 (11.3)	0.12
Type of first LRE, n (% to all LRE)			
Ascites	10 (50.0)	17 (60.7)	
Hepatic encephalopathy	4 (20.0)	5 (17.8)	
Upper gastrointestinal bleeding	1 (5.0)	0 (0)	
HCC	5 (25.0)	6 (21.4)	
CV events, n (%)	6/271 (2.2)	3/262 (1.1)	0.24
Type of CV event, n (% to all CV)			
Cerebrovascular	1 (16.6)	1 (33.3)	
Ischemic heart disease	3 (50.0)	1 (33.3)	
Others	2 (33.3)	1 (33.3)	
Mortality, n (%)	9/271 (3.3)	16/262 (6.1)	0.12
Cause of death, n (% to all death)			
Liver-related	5 (55.5)	1 (6.2)	
CV	0	1 (6.2)	
Extrahepatic cancer	1 (11.1)	2 (12.5)	
COVID-19	0	9 (56.2)	
Other	3 (33.3)	3 (18.7)	
Composite endpoint (any clinical outcome), n (%)	27/271 (9.9)	44/247 (17.8)	0.009

All comparisons were performed using the test on the equality of proportions. A *p* <0.05 was considered statistically significant.

CV, cardiovascular; HCC, hepatocellular carcinoma; LRE, liver-related event.

No statistical differences were found when comparing the incidence of CV events between study subperiods (6/271 *vs.* 3/262; *p =* 0.24). The overall mortality rate before the pandemic outbreak was 3.3%, whereas it was 6.1% (16/262) during the Pandemic period (*p =* 0.12). Of note, during the latter 9/16 deaths were due to COVID-19.

**Fig. 1:**
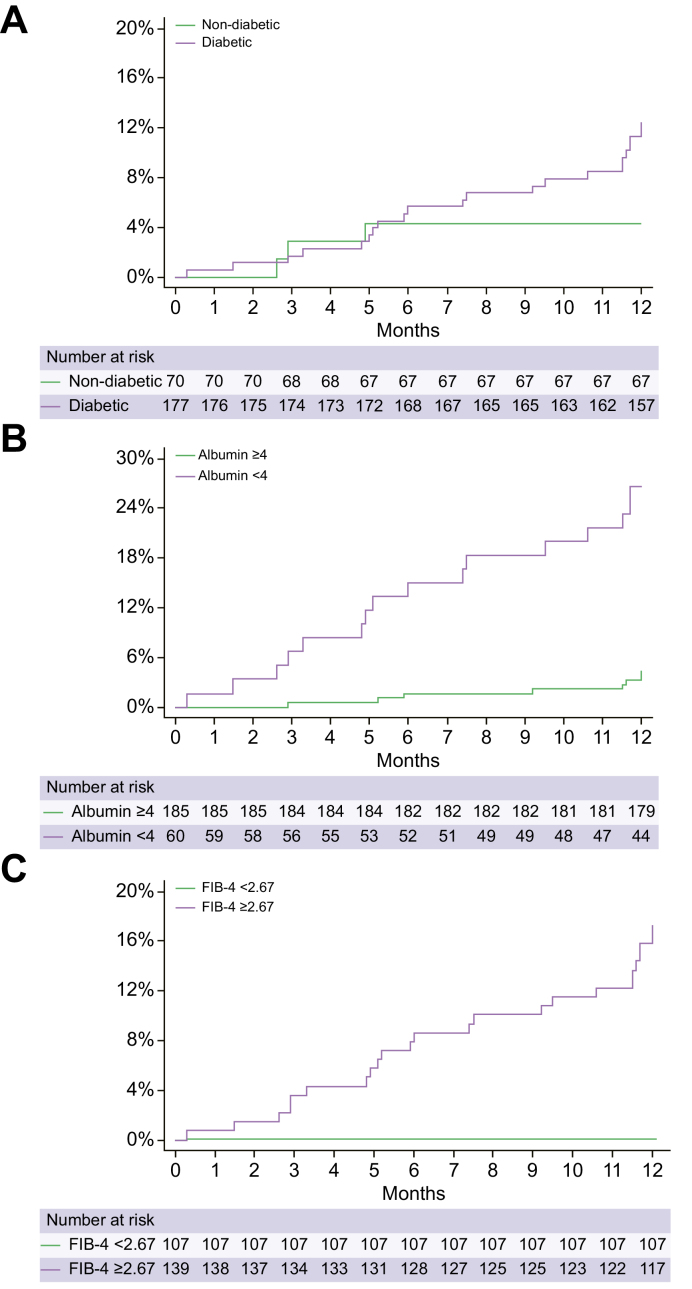
Kaplan-Meier survival curves showing first liver-related events during the Pandemic period in people with compensated NAFLD cirrhosis without prior decompensations (n = 271). (A) By type 2 diabetes status (log-rank test = 0.08); (B) albumin serum levels ≥4 (log-rank test <0.001); and (C) FIB-4 score ≥2.67 (log-rank test <0.01). The equality of survivor functions was tested with the log-rank test and a *p* <0.05 was considered statistically significant. FIB-4, Fibrosis-4; NAFLD, non-alcoholic fatty liver disease.

Meanwhile, the incidence of overall events (LRE, CV event, and/or death from any cause) during the Pandemic period was significantly higher than that of the Pre-Pandemic period (17.8% *vs.* 9.9%, respectively; *p =* 0.009). The cumulative incidence of first LRE is shown in Fig. 1.

### Secondary outcomes amongst the entire cohort of people with compensated NAFLD cirrhosis

As secondary outcomes, we assessed changes in the metabolic status, potential deferrals in the management of cirrhosis hallmarks (*i.e.*, esophageal varices or HCC) and the occurrence of clinical events (Table 4).

**Table 4:** **Metabolic and clinical outcomes before and after the outbreak of the COVID-19 pandemic**.

Outcomes	Overall, n = 354			Without prior decompensation, n = 271			With prior decompensation(s), n = 83		
	Pre-pandemic	Pandemic	*p* value	Pre-pandemic	Pandemic	*p* value	Pre-pandemic	Pandemic	*p* value
Metabolic status									
Significant weight gain, n (%)	40/331 (12.0)	37/247 (14.9)	0.31	32/259 (12.3)	30/197 (15.2)	0.37	8/72 (11.1)	7/50 (14.0)	0.63
Poor control of T2D, n (%)	49/353 (13.8)	44/286 (15.3)	0.59	39/271 (14.4)	38/224 (16.9)	0.43	10/82 (12.2)	6/62 (9.6)	0.63
Poor control of arterial hypertension, n (%)	13/354 (3.6)	20/301 (6.6)	0.08	11/271 (4.0)	15/251 (6.0)	0.31	2/83 (2.4)	5/70 (7.1)	0.06
Poor control of dyslipidaemia, n (%)	80/354 (22.6)	76/333 (22.8)	0.94	68/271 (25.1)	65/261 (24.9)	0.96	12/83 (14.4)	11/72 (15.2)	0.88
Overall worsening of metabolic status, n (%)	136/354 (38.4)	154/334 (46.1)	0.041	111/271 (40.9)	123/262 (46.9)	0.16	25/83 (30.1)	31/72 (43.0)	0.09
Delayed outcomes, n (%)	1/354 (0.3)	4/334 (1.2)	0.006	0	3/262 (1.15)	∗	1/83 (1.2)	1/72 (1.4)	0.3
Type of delayed outcomes, n (% to all outcomes)									
Delayed HCC diagnosis, n (%)	0	4 (100)	∗	0	3 (100)	∗	0	1 (100)	∗
Delayed varices treatment, n (%)	1 (100)	0	∗	0	0	∗	1 (100)	0	∗
Type of LRE									
Ascites, n (%)	36/354 (10.1)	31/309 (10.0)	0.95	12/271 (4.4)	21/255 (8.2)	0.07	24/83 (28.0)	10/54 (18.5)	0.16
HE, n (%)	29/354 (8.1)	23/315 (7.3)	0.66	9/271 (3.3)	10/257 (3.8)	0.72	20/83 (24.1)	13/58 (22.4)	0.81
Upper gastrointestinal bleeding, n (%)	8/354 (2.2)	8/327 (2.4)	0.87	3/271 (1.1)	3/260 (1.1)	0.91	5/83 (6.0)	5/62 (7.4)	0.63
SBP, n (%)	4/354 (1.1)	7/333 (2.1)	0.21	2/271 (0.7)	3/262 (1.1)	0.43	2/83 (2.4)	4/71 (5.6)	0.09
HCC, n (%)	9/354 (2.5)	9/326 (2.7)	0.85	5/271 (1.8)	8/257 (3.1)	0.34	4/83 (4.8)	1/69 (1.4)	0.02
Liver transplant, n (%)	0	1 (100)	∗	0	1 (100)	∗	0	0	∗
Total individuals with any LRE, n (%)	59/354 (16.7)	65/334 (19.4)	0.34	20/271 (7.3)	35/262 (13.3)	0.02	39/83 (46.9)	30/72 (41.6)	0.50
CV events, n (%)	9/354 (2.5)	6/334 (1.8)	0.5	6/271 (2.2)	3/262 (1.1)	0.24	3/83 (3.6)	3/72 (4.1)	0.8
Type of CV event, n (% to all CV)									
Stroke	1 (11.1)	1 (16.6)		1 (16.6)	1 (33.3)		0	0	
Ischemic heart disease	5 (55.5)	1 (16.6)		3 (50.0)	1 (33.3)		2 (66.6)	0	
Other	3 (33.3)	4 (66.6)		2 (33.3)	1 (33.3)		1 (33.3)	3 (100)	
Mortality, n (%)	20/354 (5.6)	28/334 (8.3)	0.15	9/271 (3.3)	16/262 (6.1)	0.12	11/83 (13.2)	12/72 (16.6)	0.55
Cause of death, n (% to all death)									
Liver-related	10 (50.0)	7 (25.0)		5 (55.5)	1 (6.2)		5 (45.4)	6 (50)	
CV	2 (10.0)	2 (7.1)		0	1 (6.2)		2 (18.1)	1 (8.3)	
Extrahepatic cancer	1 (5.0)	3 (10.7)		1 (11.1)	2 (12.5)		0	1 (8.3)	
COVID-19	0 (0)	9 (32.1)		0	9 (56.2)		0	0	
Other	7 (35.0)	7 (25.0)		3 (33.3)	3 (18.7)		4 (36.3)	4 (33.3)	
Composite endpoint (any clinical outcome), n (%)	71/354 (20.0)	82/334 (24.5)	0.15	29/271 (10.7)	49/262 (20.0)	0.009	42/83 (50.6)	33/72 (45.8)	0.55

All comparisons were performed using the test on the equality of proportions. A *p* <0.05 was considered statistically significant.

CV, cardiovascular; HCC, hepatocellular carcinoma; HE, hepatic encephalopathy; LRE, liver-related event; SBP, spontaneous bacterial peritonitis; T2D, type 2 diabetes.

Although no significant differences were found in individual metabolic comorbidities between both subperiods, the rate of worse overall metabolic status was significantly higher in the Pandemic period (38.4% *vs.* 46.1% *p =* 0.041). No differences were found regarding HCC diagnostic delay and esophageal varices treatment.

No differences were found between periods when comparing the global number of patients that presented with any type of LRE nor by specific decompensating event. One patient underwent a liver transplant during the Pandemic period. The baseline characteristics of the entire study cohort according to Child-Pugh classification (A *vs.* B–C) are provided in the supplementary information.

### Multivariable analysis of risk factors for LRE

Finally, we performed a logistic regression analysis to identify predictors of presenting a first LRE amongst people with cirrhosis without prior decompensations during the Pandemic period. As shown in Table 5, worsening of metabolic status during the Pandemic period was not associated with the development of a first LRE. However, T2D (odds ratio [OR] 3.77; 95% CI 1.15–12.32; *p =* 0.028), albumin <4 g/L (OR 4.43; 95% CI 1.76–11.17; *p =* 0.002) and FIB-4 score >2.67 (OR 15.74; 95% CI 2.01–123.22; *p =* 0.009) were identified as risk factors for a first LRE in the multivariable analysis.

**Table 5:** **Risk factors associated with the development of a first liver-related event during the pandemic period amongst people with NAFLD cirrhosis without prior decompensations**.

	Univariate regression			Multivariable regression		
	OR	95% CI	*p* value	OR	95% CI	*p* value
Age	0.99	0.92-1.08	0.96			
Female sex	3.21	0.82-1.03	0.09	0.49	0.19-1.23	0.13
Arterial hypertension	1.03	0.28-3.74	0.95			
T2D	0.10	0.01-0.52	0.007	3.77	1.15-12.32	0.028
Dyslipidaemia	1.95	0.67-6.17	0.25			
Metabolic status worsening	2.65	0.78-8.88	0.11			
BMI	0.92	0.82-1.03	0.16			
Creatinine	1.88	0.12-28.77	0.64			
Albumin∗	0.21	0.06-0.73	0.014	4.43	1.76-11.17	0.002
Bilirubin	0.62	0.13-2.86	0.54			
MELD score	0.87	0.61-1.24	0.46			
FIB-4 score∗∗	1.49	1.18-1.89	0.001	15.74	2.01-123.22	0.009

Results from univariate and multivariable regression analysis. A *p* <0.05 was considered statistically significant.

MELD, model for end-stage liver disease; NAFLD, non-alcoholic fatty liver disease; OR, odds ratio; T2D, type 2 diabetes.

∗ Albumin cut-off <4.0 g/dl.

∗∗ FIB-4 score cut-off >2.67.

## Discussion

In the present study, we analysed a well-characterized multicentric cohort to investigate the impact of the COVID-19 pandemic on a particularly vulnerable population, namely people with cirrhosis due to NAFLD. Three hundred and fifty-four people with NAFLD cirrhosis were evaluated during two subperiods, from March 2019 to February 2020 (Pre-pandemic period) and between March 2020-February 2021 (Pandemic period). We observed that the proportion of people with compensated NAFLD cirrhosis presenting any clinical outcome (liver, CV event and/or death due to any cause) during the Pandemic period was higher than in the pre-Pandemic period, however this was due mostly to non-liver events and in particular to COVID-19 deaths. Moreover, worsening of metabolic status was not identified as a risk factor for a first cirrhosis decompensation.

The primary outcome we investigated was the incidence of a first LRE amongst people with compensated cirrhosis, since they comprise the bulk of NAFLD cirrhosis globally and therefore our findings could have informed strategies to prevent hepatic decompensation. In addition, we hypothesized that an overall lack of physical exercise, poor diet adherence, alcohol consumption, weight gain and psychological distress during the first year of the pandemic might have led to worsening of metabolic status in a significant proportion of patients, and this could be a major trigger of first LRE. However, we found that the incidence of LRE was similar between periods in compensated patients. No significant differences were found between periods when analyzing the incidence of LRE, CV events and mortality separately in the entire cohort (*i.e*., also including patients previously decompensated at baseline). Conversely, a significantly higher proportion of the overall cohort presented impaired metabolic control during the pandemic. However, we did not find an independent association between a first LRE and metabolic worsening, which is in disagreement with prior reports.^17^^,^^18^^,^^29^ We believe this could be partially explained by the relatively small number of events occurred during the study period and also because although metabolic status worsened overall none of its components separately worsened in a significant manner. Further prospective studies that systematically collect metabolic data on people with NAFLD and evaluate the longitudinal changes along the Pandemic period are needed.

On the other hand, when analyzing the occurrence of any clinical outcome together (LRE, CV and/or death) we observed that compensated patients were more likely to present an event during the pandemic with respect to the Pre-Pandemic period. This is in line with the observed worsening in liver function and renal parameters, which are well-known predictors of hepatic and extrahepatic events in cirrhosis, including NAFLD.^30^ Yet, two observations prevent us from drawing clear conclusions. First, worsening liver and renal parameters mostly relied on previously decompensated patients, which is consistent with the natural history of the disease and might not be associated with the pandemic. Second, if it were not for the 9 deaths due to COVID-19, the rates of overall events would not have reached statistical significance and would actually have been similar. Therefore, we cannot conclude that the first year of the pandemic and its potentially associated factors had a strong impact on NAFLD outcomes other than the mortality induced by the viral infection itself, as previously described.^6^^,^^7^ We believe that the enormous effort of all the healthcare professionals in these hospitals ensured that a high-quality clinical service was maintained for people with cirrhosis. This is supported by the lack of differences in delayed diagnostic and therapeutic measures between the two periods.

We found that T2D, albumin levels and FIB-4 score were independently associated with the development of a first LRE in compensated patients during the first year of the COVID-19 pandemic. Our results are consistent and reproduce previous findings, where metabolic comorbidities, decline in serum albumin concentration and serologic non-invasive tests have proven to predict clinical events in people with cACLD due to NAFLD.[30], [31], [32]

Our results underscore the vulnerability of people with NAFLD cirrhosis and the importance of the healthcare system, from primary care to liver clinics, in their care. In order to avoid deleterious impacts of future healthcare crises, whatever the cause, healthcare providers and policymakers, alongside the patients and their communities, should advocate for health educational programs, community health interventions including screening and early diagnosis, e-health systems, and other measures that make people with cirrhosis less dependent on specialized care. Liver specialists should continue to play a key role in the follow-up and management of these patients, but sustainable models for both the patients, the healthcare systems and the taxpayers that rely on transversal multidisciplinary teams are increasingly necessary to cope with the mounting complexity surrounding the care of people with cirrhosis. Meanwhile, contingency plans to face further pandemic waves, relying on a smooth coordination between the primary and the tertiary setting and on improved referral pathways, are essential.

Our study is constrained by several limitations. First, the low number of clinical events, likely determined by the short study period and the sample size analyzed. On the other hand, it is worth highlighting that the first and second COVID-19 waves (from March to December 2020, approximately) were particularly intense in Catalonia. Consequently, the overwhelmed healthcare system missed relevant information regarding non-fatal events or metabolic status during several months, thus likely leading to an underestimation of events. Moreover, information regarding non-invasive tests such as VCTE in the Pandemic period is limited due to restriction in routine tests until the end of 2020, hampering the utilization of liver stiffness data in the analyses of risk factors of first LRE.

In our study, people with cirrhosis due to NAFLD did not present a higher rate of LREs during the first year of the COVID-19 pandemic. Diabetes, lower albumin and higher FIB-4 were associated with a higher risk of a first LRE. Longitudinal studies with larger sample sizes are needed to assess the specific impact of the pandemic on people with NAFLD cirrhosis. Regardless of the epidemiological situation, it is fundamental to ensure a proper surveillance of people with cirrhosis and early management of complications.

## Financial support

No funding was obtained to carry out the present work.

## Authors’ contributions

Conceptualization and design: JRE, RM, JMP; Data collection: JRE, RM, TB, ISM, OB, ASV, GE, JC, JB; Drafting of first manuscript: JRE, JMP; Data analyses: JRE, RM; Data interpretation: JRE, RM, TB, JCar, PG, IG, JMP; Critical revision of the manuscript: RM, TB, ISM, OB, ASV, GE, JC, JB, PG, IG; Supervision: TB, ISM, PG, IG, JMP; Access and verification of data: JMP. All authors confirm that they had full access to all the data in the study and accept responsibility to submit for publication.

## Data availability statement

De-identified data will be shared upon request.

## Conflicts of interest

TB has received educational and research support from Gilead and Abbvie. JMP reports having received consulting fees from Boehringer Ingelheim and Novo Nordisk. He has received speaking fees from Gilead, and travel expenses from Gilead, Rubió, Pfizer, Astellas, MSD, CUBICIN, and Novo Nordisk. He has received educational and research support from Gilead, Pfizer, Astellas, Accelerate, Novartis, Abbvie, ViiV, and MSD, and funds from European Commission/EFPIA IMI2 853966-2, IMI2 777377, H2020 847989, and ISCIII PI19/01898. Other authors: nothing to disclose. None of the authors have any personal conflict with regards to the present manuscript.

Please refer to the accompanying ICMJE disclosure forms for further details.
