# Supplementary material for: Impact of the COVID-19 pandemic on the care and outcomes of people with NAFLD-related cirrhosis
Source: JHEP Rep. 2022 Aug 27;4(11):100574. doi: 10.1016/j.jhepr.2022.100574 (PMC9419430; doi:10.1016/j.jhepr.2022.100574)
Supplement: Multimedia component 1 [file mmc1.pdf]

Impact of the COVID-19 pandemic on the care and outcomes of patients with NAFLD-related cirrhosis

Jesús Rivera-Esteban, Ramiro Manzano-Nuñez, Teresa Broquetas, Isabel Serra-Matamala, Octavi Bassegoda, Agnès Soriano-Varela, Gemma Espín, Joaquín Castillo, Juan Bañares, José Carrión, Pere Ginès, Isabel Graupera, Juan M Pericàs

Table of contents

Table S1..2

Table S1. Baseline characteristics of the cohort according to Child-Pugh classification.

	**Overall**<br>**n=354**	**Child A**<br>**n=287**	**Child B-C**<br>**n=43**	**p value**
**Age, mean years (SD)**	67.3 (9.6)	66.9 (9.2)	68.8 (10.5)	0.11
**Females, n (%)**	171 (48.3)	138 (48.1)	19 (44.2)	0.63
**Tobacco use, n (%)**	38 (10.8)	32 (11.1)	3 (7.0)	0.21
**Alcohol use, n (%)***	76 (21.5)	62 (21.6)	11 (25.6)	0.57
**Body Mass Index, median kg/m^2 (IQR)**	31.2 (27.6-35.1)	31.8 (27.8-35.3)	30.2 (26.9-32.9)	0.023
**BMI $\geq$ 25 kg/m^2, n (%)**	302 (92.9)	249 (92.9)	35 (94.6)	0.70
**BMI $\geq$ 30 kg/m^2, n (%)**	188 (57.8)	152 (56.7)	23 (62.2)	0.53
**Arterial hypertension, n (%)**	251 (70.9)	206 (71.8)	29 (67.4)	0.55
**T2D, n (%)**	258 (72.9)	214 (74.6)	28 (65.1)	0.19
**Dyslipidaemia, n (%)**	181 (51.1)	156 (54.4)	15 (34.9)	0.017
**Previous stroke, n (%)**	16 (4.5)	12 (4.2)	3 (7.0)	0.41
**Previous ischemic heart disease, n (%)**	35 (9.9)	31 (10.8)	3 (7.0)	0.44
**Liver stiffness, mean kPa (SD)****	23.6 (14.8)	22.9 (13.8)	33.9 (25.4)	0.10
**CAP, mean dB/m (SD)*****	307.0 (58.0)	308.1 (57.6)	281.3 (77.6)	0.43

*Alcohol intake was defined as <20 gr/day and <30gr/day for women and men, respectively.
*Data available in 83 subjects.
**Data available in 75 subjects.
